# Characterization of Circadian Behavior in the Starlet Sea Anemone, *Nematostella vectensis*

**DOI:** 10.1371/journal.pone.0046843

**Published:** 2012-10-09

**Authors:** William D. Hendricks, Christine A. Byrum, Elizabeth L. Meyer-Bernstein

**Affiliations:** Department of Biology and Program in Neuroscience, College of Charleston, Charleston, South Carolina, United States of America; Vanderbilt University, United States of America

## Abstract

**Background:**

Although much is known about how circadian systems control daily cycles in the physiology and behavior of *Drosophila* and several vertebrate models, marine invertebrates have often been overlooked in circadian rhythms research. This study focuses on the starlet sea anemone, *Nematostella vectensis*, a species that has received increasing attention within the scientific community for its potential as a model research organism. The recently sequenced genome of *N. vectensis* makes it an especially attractive model for exploring the molecular evolution of circadian behavior. Critical behavioral data needed to correlate gene expression patterns to specific behaviors are currently lacking in *N. vectensis*.

**Methodology/Principal Findings:**

To detect the presence of behavioral oscillations in *N. vectensis*, locomotor activity was evaluated using an automated system in an environmentally controlled chamber. Animals exposed to a 24 hr photoperiod (12 hr light: 12 hr dark) exhibited locomotor behavior that was both rhythmic and predominantly nocturnal. The activity peak occurred in the early half of the night with a 2-fold increase in locomotion. Upon transfer to constant lighting conditions (constant light or constant dark), an approximately 24 hr rhythm persisted in most animals, suggesting that the rhythm is controlled by an endogenous circadian mechanism. Fourier analysis revealed the presence of multiple peaks in some animals suggesting additional rhythmic components could be present. In particular, an approximately 12 hr oscillation was often observed. The nocturnal increase in generalized locomotion corresponded to a 24 hr oscillation in animal elongation.

**Conclusions/Significance:**

These data confirm the presence of a light-entrainable circadian clock in *Nematostella vectensis*. Additional components observed in some individuals indicate that an endogenous clock of approximately 12 hr frequency may also be present. By describing rhythmic locomotor behavior in *N. vectensis*, we have made important progress in developing the sea anemone as a model organism for circadian rhythm research.

## Introduction

Throughout the natural world, rhythms in behavior and physiology have been observed in most organisms, including eukaryotes, prokaryotes, and even in members of the *Archaea*. The range of frequencies in which these biological oscillations are expressed within an individual can be quite diverse and is likely to depend on the organism’s specific needs for survival. The spectrum of rhythms includes ultradian (minutes), circatidal (12.4 hrs), circadian (24 hrs), circalunar-day (24.8 hrs), semilunar (15 days), lunar (28 days), or circannual (yearly) oscillations [1], [2], [3], [4]. Among these, the most widely studied is the circadian rhythm, a cyclic pattern that has been documented in most species studied to date. Undoubtedly, the ubiquitous presence of the circadian rhythm is an indication of the evolutionary significance of this particular temporal oscillation.

Although regulation of circadian cycles has been thoroughly investigated in some derived metazoan taxa, much remains to be learned about circadian control in pleisiomorphic groups such as the Cnidaria. In this phylum, which includes organisms such as sea anemones, corals, jellyfish, and *Hydra*, diel patterns of behavior have been documented in species of the subphyla Anthozoa [5], [6], [7], [8], [9], [10], [11], [12], [13] and Medusozoa [14], [15], [16], [17], [18], [19], [20], but only a few investigators have attempted to discern whether these behaviors are direct responses to changes in light availability or whether the behavior is endogenously generated by a circadian clock. Depending on the species tested and the environmental conditions, studies have reported the presence of behaviors dependent on an internal clock, as well as behaviors that cycle merely in response to the exogenous photoperiod. Specifically, in some of the earliest studies, Bohn [5] found that retraction and expansion of the body column in the sea anemone *Actinia equina* followed a daily pattern that persisted in constant darkness for 3 to 8 days in the laboratory. Similar observations were made in the sea anemone *Metridium senile* [6] and the sea pen *Cavernularia obesa* [7], [8]. In contrast, an early study by Parker [5] reported that the sea anemone *Metridium marginatum* exhibited daily oscillations in expansion and retraction that failed to persist under constant dim lighting. These inconsistencies have also been observed in some coral species. The comparative work of Sweeney [9] looked at circadian behaviors in 21 coral species. In most corals studied, tentacle expansion fluctuated daily. Typically, individuals were nocturnal, extending their tentacles at night and retracting them during the day. When exposed to continuous darkness, only the fungids continued to exhibit rhythmic patterns of contraction and expansion. The non-persistent behaviors reported in some of the corals and sea anemones may indicate that these measured behaviors are not under the control of a circadian clock, but this does not exclude the possibility of a circadian mechanism governing oscillations of other behaviors in these organisms. Moreover, these early studies typically utilized equipment that may not have permitted full characterization of the observed behaviors. Thus, the presence and function of an internal circadian clock within the phylum Cnidaria has not been completely resolved.

We have chosen to study circadian cycles in *N. vectensis* because this easily manipulated species has the potential to reveal the molecular basis of circadian cycles both in basal taxonomic groups and in economically desirable species such as corals. This species has become a critical model for studies in developmental biology and molecular evolution because it is quite hardy, can be induced to spawn throughout the year, and has a genome that is publicly accessible [21], [22], [23]. Also, as a cnidarian, it occupies a basal taxonomic position among metazoan phyla and, as a member of the class Anthozoa, it is pleisiomorphic within that phylum. These features make *N. vectensis* especially useful for detecting traits that may have existed among ancestral cnidarians or metazoans and, cumulatively, these characteristics make *N. vectensis* an ideal model to probe the evolution of the circadian clock.

Molecular clocks in insects and mammals have been fairly well described [24], however, only in the last few years have there been investigations into the molecular underpinnings of rhythmic behavior in cnidarians [25], [26], [27], [28]. A recent study [26] investigating circadian genes in *N. vectensis* strongly suggests that critical molecular components of the circadian clock mechanism have been conserved in these animals. Unfortunately, without clear characterization of behavior, it will be impossible to fully understand how these circadian genes function. The present study complements the molecular studies by defining locomotor behavior of *N. vectensis* across the circadian cycle. We have developed a protocol to assess locomotor behavior, setting the stage for future studies aimed at understanding the cellular and molecular control of circadian behavior in this species. In the present study, the locomotor activity of *N. vectensis* was monitored under photoperiodic and continuous light cycles. Animals exposed to a 12 hr light: 12 hr dark photoperiod were most active in the dark phase. In order to determine whether the rhythmic behavior was generated by an endogenous circadian clock or was merely a direct response to the changes in lighting, animals were exposed to conditions of continuous darkness (DD) or continuous light (LL). Cyclic patterns of locomotor activity persisted in many of these individuals, indicating endogenous circadian regulation. Also, under constant conditions, some individuals exhibited a secondary activity component. These observations suggest that one or more endogenous pathways function to regulate patterns of locomotor activity in the sea anemone *N. vectensis*.

## Results

### Entrainment: Evidence that Locomotor Activity can be Synchronized by Photic cues

**Figure 1:**
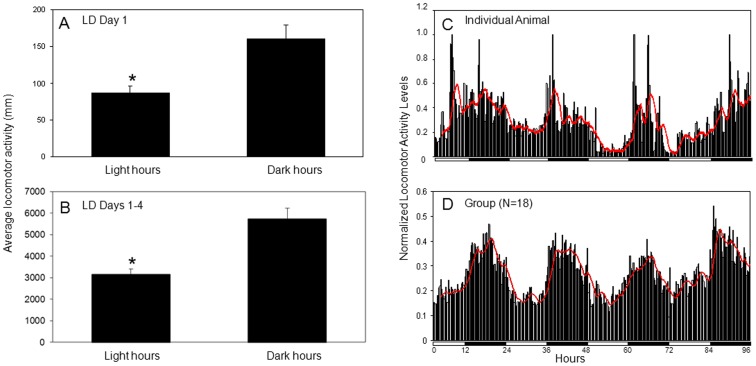
Animals housed in a 12 hr light: 12 hr dark photoperiod (LD) exhibit synchronized rhythmicity of locomotor activity. A) Average locomotor distance (mm) traveled on Day 1 of LD experiment. The total activity counts were significantly higher during the 12 hr period that animals were exposed to the dark (*t-test, *p*<0.0001) than during the 12 hr period that they were exposed to light. Error bars represent standard error. B) Average locomotor distance (mm) traveled between Days 1–4 of LD experiment. C) Normalized locomotor activity of an individual animal and D) normalized data averaged from 18 animals over the course of 4 days in 12 hr: 12 hr LD conditions. Dark bars represent the time of day when the lights were off. Locomotor activity increased during scotophase and tapered off at dawn. The red line indicates a calculated moving average of ten 15 min bins.

Under 12 hr light: 12 hr dark conditions (LD), *N. vectensis* demonstrated stable nocturnal rhythmic behavior consistent with synchronization, or entrainment, to a 24 hr photoperiod (Figure 1, S1). In 16 of 18 animals, levels of locomotor activity were significantly higher during the 12 hr night on Day 1 of the experiment as compared to the day (t(34) = 3.6, *p* = 0.001) (Figure 1A). The remaining individuals (2 of 18) appeared to be more active at night on Day 1, but in these cases the day versus night activity levels were not statistically significant. Group analysis (N = 18) revealed that over the duration of the 4-day experiment, overall locomotor activity was greater during the dark phase (scotophase) (t(34) = 4.5, *p* = 0.00007) (Figure 1B) and, on average, 65% of the locomotor activity occurred during scotophase.

In LD conditions, animals also showed anticipation of lights off, with an average increase in locomotor activity beginning approximately 40 minutes prior to dark onset (42±24 min). In 4 of the 18 animals, onset occurred much earlier, 2–3 hrs before lights off. To evaluate anticipatory activity, we summed the activity during the last 6 hours of the light period (ZT6–12) and compared the percentage of the activity that occurred during ZT6–9 to that which occurred in the 3 hours prior to lights off (ZT9–12) on the first full day of LD. We found that a significantly greater proportion of locomotion occurred in the 3 hours proceeding dark onset (55±2.9 vs. 45±2.9%) (t(34) = 2.3, *p* = 0.022). Average activity offset was 15±16 min after lights on. With one exception, the nocturnal increase in activity did not extend more than one hour past lights on in the morning. In some individuals, amplitude of the rhythm damped over the course of the experiment, but on the last day, a significant nocturnal increase in locomotion persisted in 13 of the 18 animals. This damping is likely due to the absence of food (animals were not fed during this experiment) and not a reduction in clock amplitude. We conclude this based on the observation that although animals had been maintained in the lab for several generations, most displayed robust nocturnal locomotion at the beginning of the experiment.

**Figure 2:**
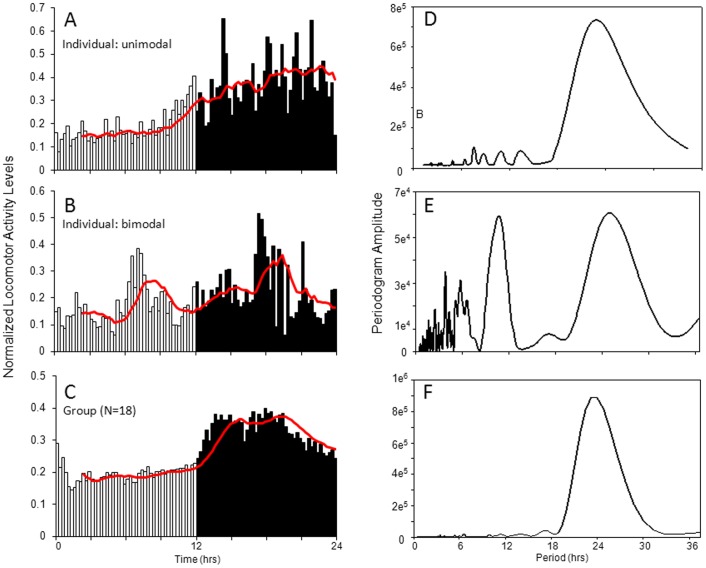
Unimodal and bimodal behavior patterns in animals housed in 12 hr light: 12 hr dark (LD). 24-hr profile of locomotor behavior in individual *N. vectensis* showing A) unimodal or B) bimodal patterns of activity. Dark bars indicate lights off and white bars indicate lights on. The red line indicates a calculated moving average of 10 bins. C) 24 hr profile of locomotor behavior averaged from 18 animals (group data) showing unimodal pattern of activity. D, E) FFT analysis of the same individuals showing a strong frequency component at approximately 24 hours. E) Example of FFT analysis from an individual showing a prominent bimodal pattern of activity with a higher frequency peak at approximately 11 hours. F) FFT analysis of group data showing a strong frequency component at approximately 24 hours. All FFT analyses were conducted on raw data over the 4 days in LD.

To identify rhythmic periodicity, the behavior of each animal was analyzed over the 4 day period in LD for frequency components using a single series Fourier analysis (Figure 2). Of the 18 animals monitored under LD conditions, a strong circadian component was detected in 17 animals with an average circadian period of 24.00±0.32 hrs and a range of 22–26 hrs. In 45% of the animals, the FFT analysis indicated a single, unimodal peak (Figure 2A,D). The Fourier analysis (FFT) was repeated on average group activity data from all 18 animals (Figure 2F) and the circadian period was calculated to be 23.97 hrs, consistent with photoperiod entrainment.

In analyzing the periodograms, we found that in 10 of the 18 animals (55%), the periodogram indicated that a higher frequency rhythm in locomotor activity was also present. In 30% of these animals, the periodogram peak was of greater amplitude than the circadian peak in the FFT analysis. These animals showed a brief rise in daytime activity, thus, yielding a bimodal distribution in behavior and periodicity (Figure 2B,E, S1, S2). Although the primary component was circadian in 70% of the animals, in half of these animals the additional component showed a peak at least half the amplitude of the circadian peak (Figure 2E). In the 10 animals analyzed, the average period of this higher frequency component was 11.00±0.36 hrs. Since this component was only present in a portion of the population, the higher frequency component is not evident in the combined group analysis, but is only obvious in the FFT analysis of individual animals (Figure 2E,F).

**Figure 3:**
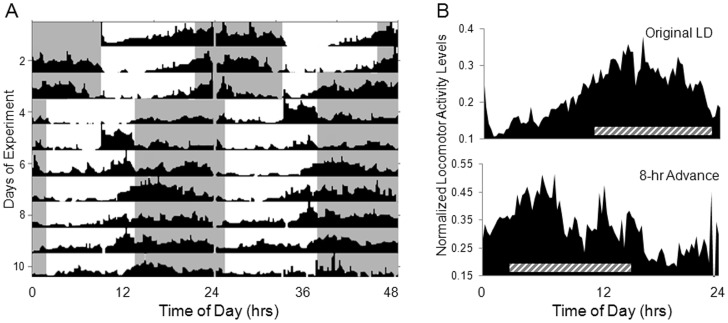
Locomotor profile shifts in response to an 8 hr advance in photoperiod. A) Actogram of averaged locomotor activity of *N. vectensis* (N = 27) over the course of the experiment. Shaded area indicates time of lights off. B) Locomotor activity profile of animals housed for 3 days in the original LD photoperiod (top) and after 7 days in an 8-hour advanced photoperiod (bottom). Seven days following the 8-hour advance in photoperiod, animals show clear entrainment to the new photoperiod. Animals exhibit an anticipatory increase in activity prior to lights off and a shift of activity to occur during the scotophase of the new LD cycle. Shaded bar represents time of lights off.

To confirm that the animals are responding to photoperiodic input, we tested their ability to entrain to a shifted LD cycle (Figure 3). As expected, animals housed in the initial LD photoperiod demonstrated an increase in locomotor activity during the nighttime hours with an advanced phase angle of entrainment similar to that reported above (average 1.58±0.5 hrs). After animals were entrained, the photoperiod was advanced by 8 hours. This was accomplished by turning the lights off 8 hrs early on Day 4 and continuing with this new LD cycle for the remainder of the experiment. After 7 days in the advanced photoperiod, the sea anemones showed a clear entrainment with an advanced onset similar to that shown in the original LD cycle (1.62±1.1 hrs prior to lights off) (Figure 3B).

### Free-running Studies: Evidence for an Endogenous Clock

**Figure 4:**
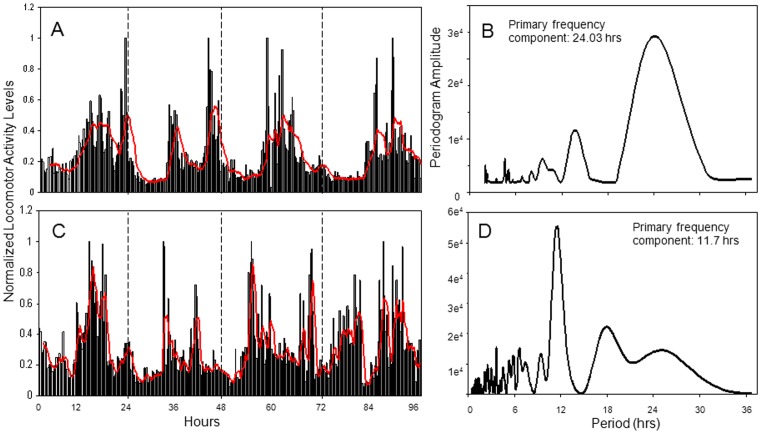
Locomotor periodicity persists in individual *N. vectensis* exposed to constant darkness. A and C) Locomotor activity of individual animals over the course of 4 days. On day 1, animals were maintained on a 12 hr: 12 hr LD photoperiod and released to constant darkness for days 2–4 of the experiment. B and D) FFT analysis was conducted on raw activity data during the DD days for each individual. B) An example of an animal with a prominent circadian component showing a period of approximately 24 hours. D) An example of an animal with a primary frequency component of approximately 12 hours. This component is not seen on Day 1 when the animal is in LD (only a nocturnal peak is evident), but emerges as the animal is transferred to constant darkness.

The ability of a rhythm to persist in the presence of constant environmental conditions is a hallmark characteristic of an endogenous oscillator. Thus, in order to determine whether the rhythmic locomotor activity observed under LD conditions could be attributed to an endogenous circadian clock, we monitored the behavior of *N. vectensis* under brief (3–4 day) constant dark conditions (Figure 4). Locomotor activity of each animal was recorded over a 24 hr LD period prior to exposure to constant dark (DD). A significant increase in nocturnal activity during LD (a t-test was carried out for each individual, *p*<0.05), and thus entrained to the LD cycle, was used as criteria to include in subsequent behavioral analysis determining rhythms sustainability. Because the sampling period was brief, these experiments were not used to predict an accurate measurement of period length. Despite this limitation, cosinor analysis was used to determine whether individual animals demonstrated a significant daily rhythm under constant conditions.

Prior to transfer to DD conditions, 12 of the 18 sea anemones demonstrated entrainment in LD. For each individual, the percentage of nocturnal activity was approximately double the activity level observed during the day (63.1±5.5% vs. 31.2±2.2%, t(22) = 11.8, *p* = 5×10^−11^). In 9 of 12 cases, FFT and cosinor analysis of the 3 days in DD revealed a prominent daily peak in activity at approximately 24 hours (Figure 4A,B, S1). In addition, the higher frequency component was again apparent in approximately half of the animals upon transfer to constant darkness (Figure 4C,D, S1).

**Figure 5:**
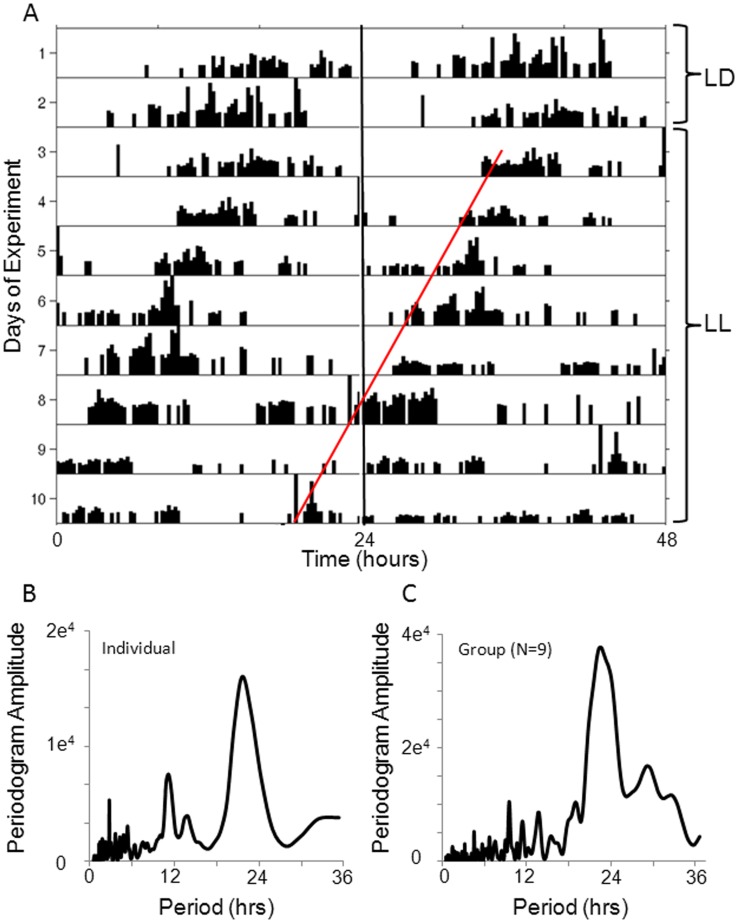
Locomotor periodicity persists in *N. vectensis* exposed to constant light. A) Actogram and B) FFT analysis of a single *N. vectensis* over the course of a 10-day experiment. Entrained animals were monitored in LD for 2 days prior to transfer to LL for a subsequent 8 days as indicated on the actogram. Locomotor activity is double plotted and a red line approximates average of activity onsets over the 8 days in LL. B) FFT analysis of this animal indicating a circadian period of 22 hours. C) FFT analysis was conducted on averaged raw activity data from all 9 animals and shows a prominent rhythm frequency approximately 22.5 hrs.

To accurately calculate the circadian period, locomotor activity was recorded from a group of entrained animals transferred into LL for 8 days (Figure 5). LL data were analyzed for rhythmic components using cosinor and FFT analysis (Figure 5B,C, S1), and a significant circadian period was detected in all animals that had demonstrated entrainment in LD (n = 7; cosinor, *p*<0.001). Of these animals, the average circadian period in LL was 23.14±0.55 hrs. Unfortunately, *ad libitum* feeding interferes with activity recording preventing data from being collected in DD for long periods of time.

Some nocturnal animals exhibit a reduction in locomotor behavior in the presence of light. This negative effect of light called “masking” can obscure overt behavioral manifestations of the clock in these organisms under certain conditions. To address this in *N. vectensis*, total activity levels during 3 days in LL or DD were compared to the activity levels of the proceeding 24 hr LD period. If light suppressed activity, an overall decrease in activity levels should be observed in LL and an increase in activity should occur in DD. No significant differences in locomotor activity levels were detected after transfer into constant dark (LD 289±13 vs. DD 315±8 counts/15 min, t(286) = 1.88, *p* = 0.07) and only minor changes were seen when animals were transferred to constant light (LD 124±5 vs. LL 140±4 counts/15 min, t(351) = 1.98, *p* = 0.04). In both paradigms, activity levels increased upon transfer from LD, however, these changes were not dependent on the lighting conditions, suggesting that light does not have a prominent direct effect on activity levels in the sea anemone.

**Figure 6:**
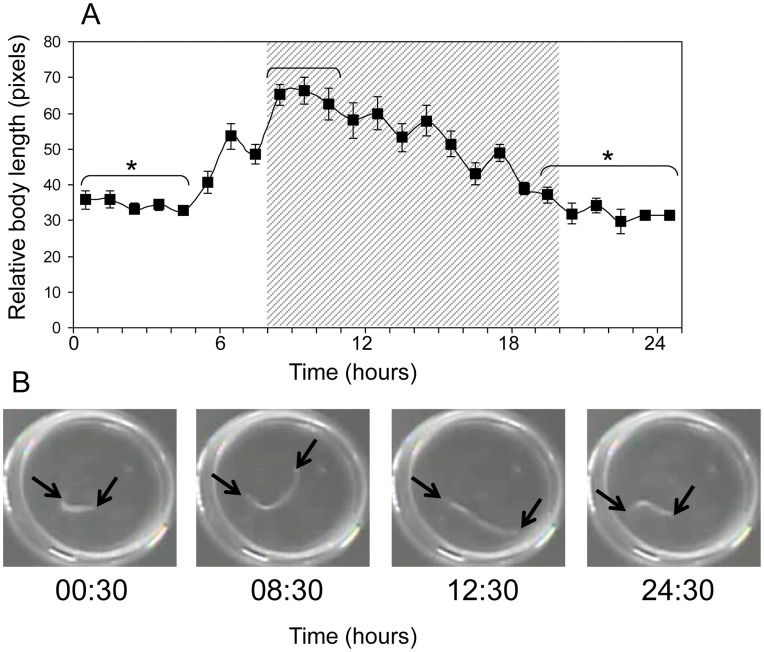
Average body length of *N. vectensis* increases in darkness. A) Average body length of 9 *N. vectensis* over a 24 hr period. Animals were housed in a 12 hr: 12 hr LD photoperiod (shaded area indicates period of lights off). Body length was significantly greater during early night as compared to time points during the day (*Newman-Keuls; p<0.05). Bars represent standard error. B) Images of an individual animal housed in a petri dish taken at various time points throughout the day. Animals remain constricted during the day and increase in length significantly during early night to an elongated, more active state. Oral and aboral ends of the animal are indicated by arrows. While peak length occurs just after lights off, animals begin to anticipate the onset of darkness by initiating elongation several hours earlier. This anticipatory behavior is characteristic of endogenous clock control.

Circadian variation in locomotor activity often reflects daily oscillations in one or more overt behaviors, such as swimming or feeding. Using twenty-four hour video of 9 entrained animals, insight into the specific behavior(s) that underlie the rhythmic observations in generalized locomotor activity was gained (Figure 6). Over the course of the day, animals undergo significant changes in body length (Figure 6A) (ANOVA, F_(24,186)_ = 5.22, *p* = 1×10^−11^). Peak extension occurred early in the night, correlating with the increase in locomotion at this time. No daily change in location within the recording dish was observed (ANOVA, F_(24,200)_ = 1.15, *p* = 0.3), suggesting that horizontal movement does not substantially contribute to the circadian rhythm in generalized locomotion.

## Discussion

By closely documenting movement in individual polyps under controlled conditions, this investigation is the first to fully describe circadian locomotor behavior in the starlet sea anemone, *N. vectensis*. Traditionally it has been difficult to perform activity assays in cnidarians. This study utilizes a new approach, an automated behavioral data collection system, to quantitatively define the locomotor activities of this marine species.

### Locomotor Activity is Regulated by a Circadian Clock

In this paper, we initially tested whether the activity patterns of *N. vectensis* could be synchronized to rhythmic daily fluctuations in illumination. We found that individual sea anemones exposed to a 12 hr light: 12 hr dark photoperiod became entrained, displaying rhythmic nocturnal increases in locomotor activity with levels of activity at night nearly double those during the day. When animals entrained to a 12 hr light: 12 hr dark photoperiod experienced an abrupt 8-hr shift in the photoperiod, they were able to adjust their activity profiles and, after a transitory period of several days, they once again displayed increased activity corresponding to the shifted dark phase. These observations indicate that locomotor behavior in *N. vectensis* can be entrained by external photoperiodic cues.

The experiments also provide evidence that an endogenous circadian clock regulates the rhythmicity of locomotor activities in *N. vectensis.* Several observations support this conclusion. First, animals entrained to a 12 hr light: 12 hr dark cycle clearly anticipated the scotoperiod by increasing activity levels prior to lights off. Thus, it was not a change in the light levels alone that influenced the timing of locomotor activity, but rather an endogenous cue. Also, many of the individuals experiencing the 8-hr photoperiod shift regained the ability to anticipate lights off after they became re-entrained to the new LD cycle. In the few transition days following the 8 hr photoperiod shift, animals displayed an increase in activity corresponding to lights off. This indicates an acute positive effect of darkness on activity levels. Based on these and previous observations, it is likely that darkness provides an independent cue to the pathways mediating activity and that changes in illumination work synergistically with the endogenous clock to optimize the timing of behavior. Finally, when animals were entrained to a 12 hr light: 12 hr dark photoperiod and then exposed to constant light or constant dark, the individuals continued to display cyclic activity patterns. Cumulatively these data provide strong evidence for the existence of an endogenous circadian clock in *N. vectensis.*

Although similar diel responses have been reported in other cnidarian species [5], [6], [7], [8], [9], [10], [11], [12], [13], most of these studies have not tested whether the behaviors persisted under constant conditions and thus, fail to demonstrate that the behaviors were controlled by an endogenous circadian rhythm. Recent studies report that several genes controlling circadian activities in vertebrates and *Drosophila* are also conserved in the cnidarian species *Nematostella vectensis* and *Acropora millepora* [25], [26], [28]. Given the conservation of circadian behaviors as well as the presence of “circadian” genes, it is likely that the common ancestors of cnidarians and metazoans possessed the capacity to regulate circadian activities. The presence of this system in such ancient species suggests that the circadian pathways are critical to the organism and act as integral components allowing the organism to anticipate changes in the external environment.

Circadian variation in the locomotor activities of this species is likely due to daily bouts of elongation and constriction. When animals were videotaped over a 24 hr period, body lengths were longer at night during periods of peak activity. It is likely that this nocturnal increase in body length correlates with feeding behavior. Previous studies have shown that elongation of the body, allowing for ingestion and peristalsis is correlated to food seeking behavior in many anthozoans and can be directly influenced by a variety of environmental stimuli [6], [8], [9]. It is likely that *N. vectensis*, similar to other animals, restricts feeding to nocturnal periods in order to reduce the chance of predation and/or to improve the chances of obtaining nocturnal prey. Differences in predator/prey distribution and zooxanthellae concentration likely account for the fact that some species of sea anemones and corals exhibit diurnal as opposed to nocturnal expansion [5], [29], [30], [31].

Another interesting feature of the locomotor behavior in *N. vectensis* is the presence of a second, higher frequency activity component in some of the animals. Approximately half of the animals expressed a higher frequency component that persisted in constant lighting conditions. Additionally, this component was more prominent in animals maintained in constant conditions as opposed to those entrained to a photoperiod. The reason for this is unknown, but may involve interaction between multiple oscillators whose phase relationships differ under photoperiodic conditions. The nature of this secondary rhythmic component is not yet clear, but the frequency of this oscillation is similar to the 12.4 hour circatidal rhythms that have been identified in other marine invertebrates including arthropods [32], [33], [34], [35], [36], [37], mollusks [38], [39] and polychaetes [4]. Further investigation leading to the establishment of endogenous circatidal and circadian behavioral oscillations may considerably enhance the value of *N. vectensis* for probing the genetic underpinnings of these behaviors and provide insight into their evolution.

### Molecular Regulation of Biological Rhythms in the Cnidaria

As mentioned earlier, several labs have started to examine the molecular components regulating patterned biological processes in cnidarians [25], [26], [28]. In comparison to what is known about the molecular clock in mammals and insects, much remains to be learned about how the clock is regulated in cnidarians. To date, studies have focused on three species, the two scleractinian corals *Acropora millepora* [25], [28] and *Favia fragum* [27] and the actiniarian *N. vectensis* [26]. In all three species, core clock components have been identified based on sequence homologies to insect and mammalian genes. *N. vectensis* expresses homologues of the circadian regulatory genes *timeout (timeless2), clock*, and *cycle (bmal)* as well as 3 different cryptochromes (*cry1a, cry1b,* and *cry2*) [26]. As noted in other model systems, several of these genes oscillate with circadian periodicity in *N. vectensis* as well as in the coral, *Favia fragum* [27]. In addition, the *Nematostella* circadian proteins, CLOCK and CYCLE, possess similar molecular characteristics to those described in other traditional model organisms [26], [40], [41]. For example, the CLOCK and CYCLE proteins hetereodimerize and both *clock* and *cry1a* possess E-box motifs to which the CLOCK/CYCLE heterodimer can bind [26]. Despite the similarities to other circadian systems, a core circadian gene, *period* (*per*), and the circadian relevant nuclear receptors ROR (NR1F1) and Rev-Erba (NR1D1) are absent in *N. vectensis* [42]. The gene product of *timeout* (*timeless2*) was detected but was not responsive to changes in light as has been shown in *Drosophila* [43]. Taken together, these studies support the hypothesis that a core group of the circadian genes in mammalian and insect models likely existed prior to the divergence of the Cnidaria from the Bilateria as evidenced by the presence of these genes in the sea anemone *Nematostella vectensis*.

It is also interesting that several rhodopsin-like G-protein coupled receptors have been identified in cnidarians [44], [45], [46]. In mammals, non-visual information is provided to the circadian clock via a G-protein coupled opsin, melanopsin [47]. Melanopsin containing cells are located in the retina, but are distinct from the processes regulating vision [48]. Rhodopsin-like receptors in cnidarians may suggest a non-visual pathway to the circadian system existed prior to the divergence of Cnidaria from the Bilateria. By learning more about the roles of opsins in cnidarian photic processes, we can gain insight into the mechanisms by which non-visual information is processed.

The ability to easily monitor behavioral rhythms and the availability of a publicly accessible, completed genome [23] makes *N. vectensis* a uniquely suited organism for the study of the molecular control of circadian systems. In addition, the presence of multiple rhythmic components in *N. vectensis* may allow for investigation of the molecular control of multiple frequency oscillations.

## Materials and Methods

### Ethics Statement

All necessary permits were obtained from South Carolina Department of Natural Resources for the field collection of *N. vectensis*. The field location is part of the Fort Johnson Marine Laboratories, a consortium of which the College of Charleston is a member. This location is not privately owned or protected and does not require any additional special permission. Collection of animals did not involve endangered or protected species.

### Husbandry

*N. vectensis* adults collected from a salt marsh pool in the Charleston, SC harbor area were maintained in the laboratory as groups in 9″ glass fingerbowls for approximately one year prior to the initiation of the experiment. Seawater (34–36 ppt) was replaced weekly and individuals were fed a diet of brine shrimp (*Artemia sp.*), mixed zooplankton (Cyclop-eeze, Age of Aquariums, Charleston, SC), and chopped mussels two times each week.

### Behavior

Prior to behavioral data collection, animals were transferred to a light (12 hr light: 12 hr dark) (approximately 450 lux, 546 nm) and temperature (25°C) controlled incubator for a minimum of 7 days in order to synchronize their behavior. For each experimental run, 9 sea anemones were placed into separate glass petri dishes (5.5×1.5 cm) containing seawater (depth 1 cm) and their locomotor behavior was recorded for the duration of the experiment. In most of the experiments, animals were fed prior to, but not during the recording period in order to eliminate any behavioral changes in response to food availability or intake. In the LL experiment, animals were hand fed mussel on LL Day 5. This did not have any measureable effect on the animal’s activity.

Locomotor activity was monitored using Noldus’s EthoVision 7 (Noldus Information Technology, Wageningen, The Netherlands) animal behavior monitoring system. Animals were visualized using a video camera mounted on a camera stand inside the incubator. In order to detect individual animals under both light and dark conditions, an infrared light source (Tracksys Ltd, Nottingham, UK) (775–100 nm, peak at 880 nm) was placed in the incubator and an IR high-pass filter restricted the detection of IR light to the camera.

Still video images of the animals (N = 9) were taken every hour for 24 hours and used to evaluate behavior. To calculate body length extension, the distance from head to tail (pixels) was measured. Horizontal movement was calculated by superimposing a 9-region grid on the recording petri dish. Grid lines crossed were tabulated for each animal throughout the day.

### Data Analysis

Data were collected as distance traveled (mm) every 5 seconds for the duration of the experiment and combined into 15-minute bins for analysis. Extreme care was taken to avoid any recording artifacts due to light reflection off of the petri dish. To eliminate these potential errors in data collection, values exceeding 1,000 mm/15 min were removed from the analysis. Data was imported into ClockLab software (Actimetrics, Wilmette, IL), Cosinor and CSS Statistica (StatSoft, Tulsa, OK) for circadian and statistical analysis. ClockLab generated actograms were used to measure individual activity onsets and offsets. Under acute exposure to constant conditions, the persistence of rhythmic behavior was evaluated with a single series spectral fast Fourier transform (FFT) analysis using CSS Statistica software. Periodicity was calculated using an online free access program (Circadian Rhythm Laboratory Software, R. Refinetti) to perform cosinor and Lomb-Scargle analysis. Limits were set to detect the significance of a rhythm in the 22–26 hr or 10–14 hr range. Periodicities were confirmed using the Chi-square analysis feature in ClockLab. For presentation, data (mm/15 min) were normalized to the maximum activity bin in a 24 hour period and plotted over the days of the experiment.

## Supporting Information

Figure S1 **Lomb-Scargle periodograms.** An additional analysis was performed on the raw data using this method and results were consistent with those reported in the manuscript that were calculated using the Chi-Square, FFT and Cosinor methods.
(TIF)

Figure S2 Actogram of locomotor activity of animals (N = 10) displaying a bimodal pattern of activity over the course of 3 days in LD. Daytime bout of activity is indicated by the star. Photoperiod is denoted by the bar at the bottom of the figure.
(TIF)
