# Supplementary figures and images for: Characterization of Circadian Behavior in the Starlet Sea Anemone, Nematostella vectensis
Source: PLoS One. 2012 Oct 9;7(10):e46843. doi: 10.1371/journal.pone.0046843 (PMC3467289; doi:10.1371/journal.pone.0046843)

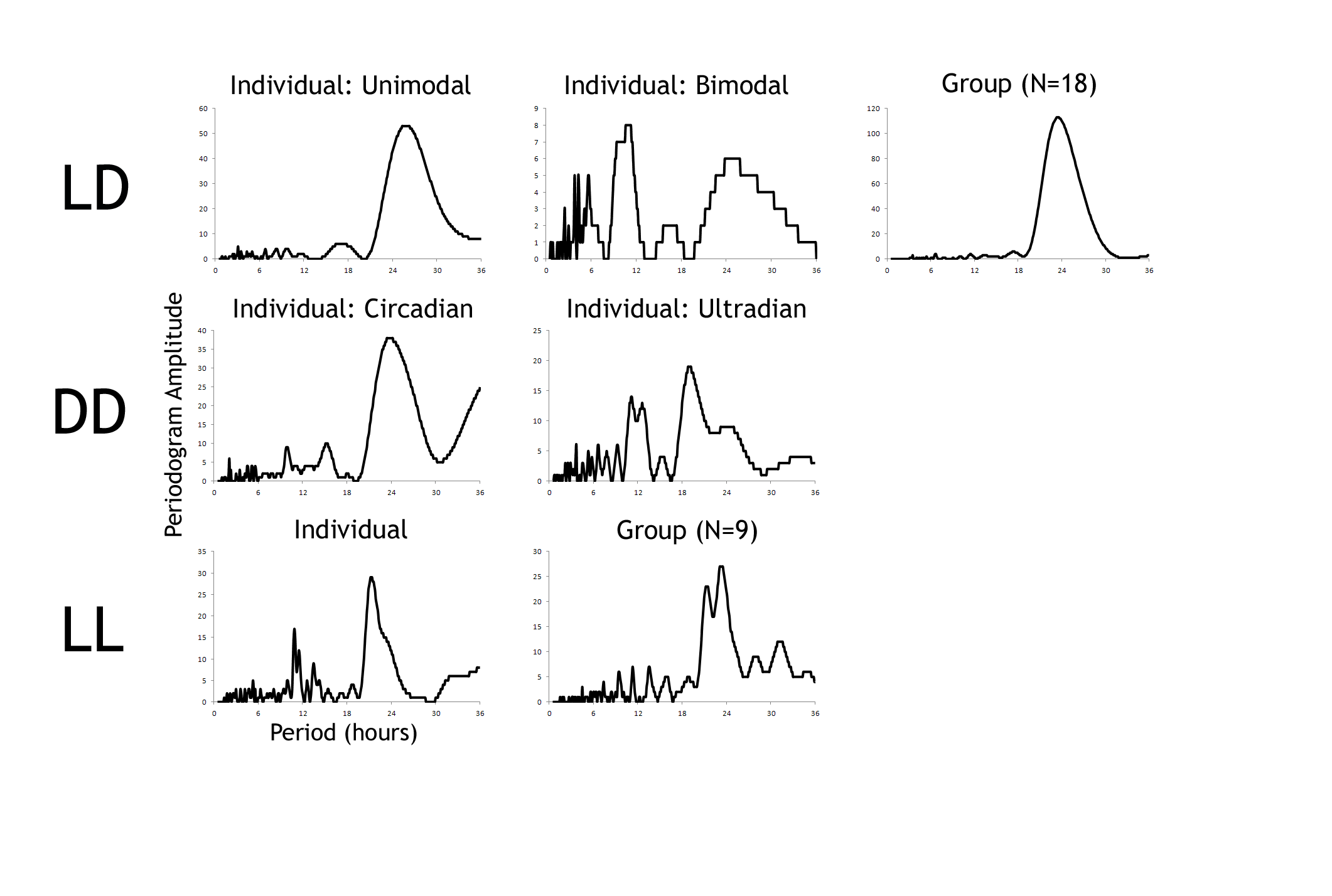

Supplement: Figure S1 — Lomb-Scargle periodograms. An additional analysis was performed on the raw data using this method and results were consistent with those reported in the manuscript that were calculated using the Chi-Square, FFT and Cosinor methods. (TIF) [file pone.0046843.s001.tif]

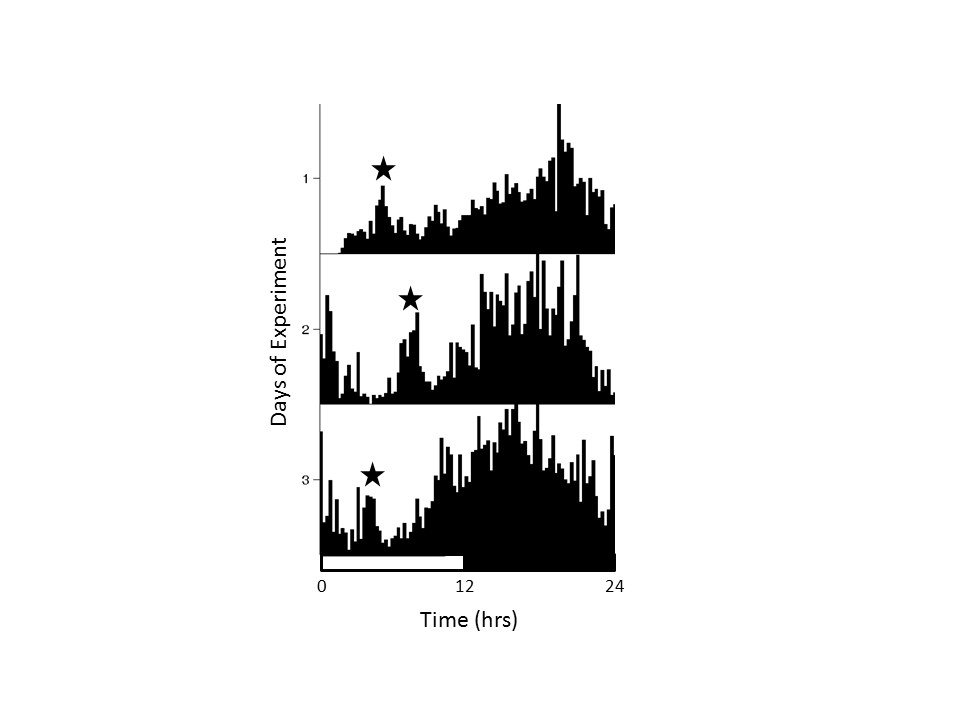

Supplement: Figure S2 — Actogram of locomotor activity of animals (N = 10) displaying a bimodal pattern of activity over the course of 3 days in LD. Daytime bout of activity is indicated by the star. Photoperiod is denoted by the bar at the bottom of the figure. (TIF) [file pone.0046843.s002.tif]
